# Evidence of probabilistic behaviour in protein interaction networks

**DOI:** 10.1186/1752-0509-2-11

**Published:** 2008-01-31

**Authors:** Joseph Ivanic, Anders Wallqvist, Jaques Reifman

**Affiliations:** 1 Biotechnology HPC Software Applications Institute, Telemedicine and Advanced Technology Research Center, U.S. Army Medical Research and Materiel Command, Ft. Detrick, MD 21702, USA

## Abstract

**Background:**

Data from high-throughput experiments of protein-protein interactions are commonly used to probe the nature of biological organization and extract functional relationships between sets of proteins. What has not been appreciated is that the underlying mechanisms involved in assembling these networks may exhibit considerable probabilistic behaviour.

**Results:**

We find that the probability of an interaction between two proteins is generally proportional to the numerical product of their individual interacting partners, or degrees. The degree-weighted behaviour is manifested throughout the protein-protein interaction networks studied here, except for the high-degree, or hub, interaction areas. However, we find that the probabilities of interaction between the hubs are still high. Further evidence is provided by path length analyses, which show that these hubs are separated by very few links.

**Conclusion:**

The results suggest that protein-protein interaction networks incorporate probabilistic elements that lead to scale-rich hierarchical architectures. These observations seem to be at odds with a biologically-guided organization. One interpretation of the findings is that we are witnessing the ability of proteins to indiscriminately bind rather than the protein-protein interactions that are actually utilized by the cell in biological processes. Therefore, the topological study of a degree-weighted network requires a more refined methodology to extract biological information about pathways, modules, or other inferred relationships among proteins.

## Background

Experimental protein-protein interaction (PPI) data and related networks, obtained from high-throughput methodology as well as hand-curation, are being widely used to probe the nature of biological organization and extract functional relationships among sets of proteins [1,2]. What has not been appreciated is that the guiding principles involved in assembling these networks may exhibit considerable probabilistic behaviour. Here, we show that the probability of an interaction between two proteins is generally proportional to the product of their individual numbers of interacting partners (or degrees) and discuss the consequences of this for probing PPI networks. Understanding the underlying organizational principles in assembling PPI networks holds the key for interpreting and analyzing the observed interactions.

High-throughput methodologies [3-6] to determine PPI networks have been used to probe the interactome of a range of organisms. The organization of these interaction networks has been studied using graph-theoretical techniques [7-9] to find global characteristics that can be mapped back to biological phenomena, such as evolutionary conserved interactions, pathway or module organization, and localization of essential proteins in the network, to mention a few. Since we know that outcomes of cellular actions are biologically "deterministic" in the sense that cells use energy, synthesize proteins, duplicate DNA, etc., the analysis of PPI networks is aimed at finding and extracting causative components. If this information is to be mined from a global dataset, it is vital to have an accurate model of the architecture of the determined PPI networks. The incorporation of the underlying determining principle of PPI organization into graph-theoretical topological studies will provide a baseline from which biologically-relevant insights could be extracted. For example, a guided biological framework implies that cellular processes consist of precise and unique protein-protein interactions, whereas a probabilistic model is suggestive of an underlying principle that is more chemical than biological, describing the *ability *of proteins to bind.

We show here that currently available PPI data support the latter interpretation and demonstrate that the probability of an interaction between two proteins is proportional to their numbers of interacting partners. The observations suggest that PPI networks are almost completely probabilistic and, therefore, in a proteome context, PPI interactions for specific biological processes are *generally *not distinct. From a purely biological point of view, the knowledge of any potential interactions between proteins is useful. However, by identifying common themes in large PPI networks, the underlying principles responsible for the discovered interactions may become more apparent.

Networks can be constructed directly from probabilistic procedures where the interactions, or edges, between two nodes is determined from an *a priori *probability distribution of edges, the simplest being the Erdös-Rényi random model [10,11]. However, biological networks, including PPI typically show power-law scaling in their degree distributions, in that the probability of any node having a given number of interactions follows a power law [12-14]. As such, the Erdös-Rényi model, which generates Poissonian degree distributions, is an unsuitable archetype for PPI networks. Networks with power-law degree distributions can be constructed using a number of techniques, including those based on preferential attachment [15,16], duplication [17-19], and hierarchical [20,21] approaches. Alternatively, the geometric random model generates networks that nearly follow a power-law distribution [22]. While each of these models may have qualitatively simulated biological networks, none have consistently and accurately reproduced properties of individual PPI networks.

Here, we describe insights into the topologies of PPI networks that should serve to enhance the development of future models. A degree-weighted network is one in which the probability of an interaction between two nodes is proportional to the product of their degrees, i.e., *P*_*ij *_∝ *k*_*i*_*k*_*j*_, where *k*_*i *_and *k*_*j *_are the degrees, or number of interactions, associated with nodes *i *and *j*, respectively [23]. A type of degree-weighted network denoted "STICKY" [24] has been proposed as a model for PPI networks on the basis of similarities in derived global, or average, network properties, e.g., graphlet frequencies and average clustering coefficients. However, this model generates far too many nodes of zero degree and is therefore an unsuitable prototype for PPI networks. It is thus of importance to both qualitatively and quantitatively ascertain the extent of degree-weighted behaviour in biological networks. Here, we explore the nature of the protein-protein connectivities more directly and conclusively demonstrate that PPI networks indeed contain degree-weighted elements.

## Results

### Probabilistic behaviour in protein interaction networks

**Figure 1:**
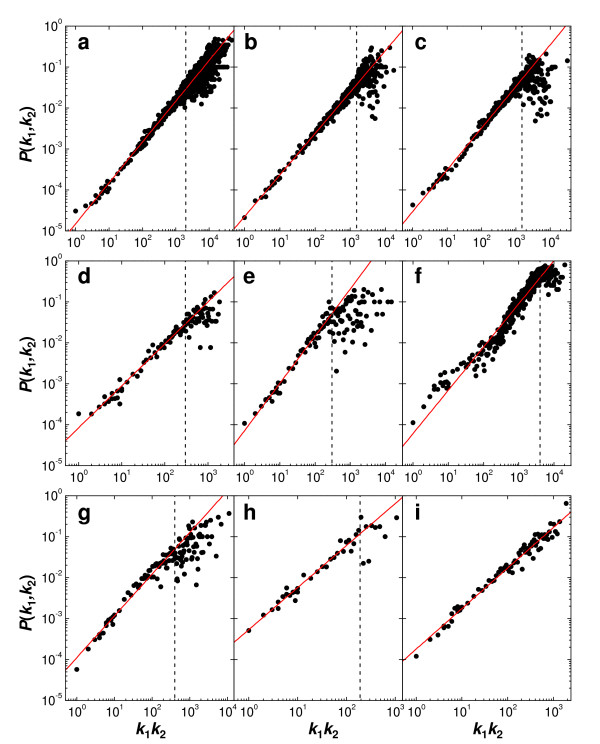
**Evidence of degree-weighted connectivity in nine PPI networks**. **a**, *Homo sapiens *(human); **b**, *Drosophila melanogaster *(fruit fly); **c-e**, *Saccharomyces cerevisiae *(yeast): Yeast-DIP, Yeast-CORE, Yeast-Y2H; **f**, *Escherichia coli *(bacterium); **g-h**, *Caenorhabditis elegans *(nematode): Worm-Y2H, Worm-CORE; **i**, *Plasmodium falciparum *(malaria-causing parasite). For *k*_1_*k*_2 _> 10, probabilities of interaction *P*(*k*_1_, *k*_2_) were ordered by *k*_1_*k*_2 _and averaged in groups of 10.

A total of nine PPI networks from six unique organisms were studied. Full details of these networks are provided in Additional file 1. Their sizes are given in Table 1. For each network we calculated the probabilities *P*(*k*_1_, *k*_2_) of interaction between two proteins of degrees *k*_1 _and *k*_2_. A probability of interaction *P*(*k*_1_, *k*_2_) is calculated by counting the total number of interactions occurring between all proteins of degree *k*_1 _and all proteins of degree *k*_2_, and dividing this by the total number of all pairs of combinations that can be made. Degree-weighted behaviour was then established by comparing the probabilities *P*(*k*_1_, *k*_2_) of interaction with the products *k*_1_*k*_2 _of the degrees (Figure 1). We find that each PPI network exhibits perfect degree-weighted behaviour up to a characteristic value of *k*_1_*k*_2_, or cutoff, which depends on the network studied. Cutoffs have been estimated for each network and these are shown as dashed lines in the graphs (Figure 1). For *Plasmodium falciparum*, degree-weighted behaviour is exemplary throughout, thus no such value could be determined. Cutoff estimates range from 200 (Worm-CORE) to 4000 (*Escherichia coli*). It is unclear why cutoff values vary greatly between networks but this is presumably related to the differences in their degree distributions in that the actual degrees of the hub proteins vary from network to network. As the number of hub proteins of a particular degree is consistently very small (one or two), one might expect more noise in the hub-hub interaction regions (largest values of *k*_1_*k*_2_). Correlation coefficients between *P*(*k*_1_, *k*_2_) and *k*_1_*k*_2 _determined using data with product degrees less than the cutoff are 0.97 or higher (Table 1), indicating an unmistakable degree-weighted signature in the PPI networks.

**Table 1:** Properties of PPI networks

Network	Number of proteins	Number of interactions	r*	*θ*^†^	*γ*^† ^(× 10^-5^)	*γ*(cal)^‡ ^(× 10^-5^)
*H. sapiens*	9263	34564	0.99	1.00 ± 0.02	1.49	1.45
*D. melanogaster*	6736	20308	0.99	1.01 ± 0.02	2.25	2.46
*Yeast*						
- *DIP*	4617	16311	0.99	1.02 ± 0.02	2.98	3.07
- *CORE*	2449	5579	0.99	1.03 ± 0.06	8.41	8.97
- *Y2H*	3277	4393	0.99	1.15 ± 0.08	7.22	11.4
*E. coli*	1473	5709	0.97	1.06 ± 0.04	6.09	8.79
*Worm*						
- *Y2H*	2624	3967	0.98	1.08 ± 0.07	9.59	12.6
- *CORE*	727	814	0.99	1.03 ± 0.09	53.5	61.7
*P. falciparum*	1304	2745	0.99	0.99 ± 0.05	18.0	18.3

*Pearson correlation coefficient for test of association between *P*(*k*_1_, *k*_2_) and *k*_1_*k*_2_, where *P*(*k*_1_, *k*_2_) is the probability of interaction between two proteins of degrees *k*_1 _and *k*_2_.

^† ^Fitted values (from the Log-Log plots in Figure 1) occurring in the expression *P*(*k*_1_, *k*_2_) = *γ*(*k*_1_*k*_2_)^*θ*^. *θ *is given with 99% confidence intervals.

^‡ ^Prediction of *γ *via *γ*(cal) = *E*/Σ_*i*<*j*_(*k*_*i*_*k*_*j*_), where *E *is the number of interactions in the network and the summation is over all pairs of proteins.

If we express the probability of interaction as *P*(*k*_1_, *k*_2_) = *γ*(*k*_1_*k*_2_)^*θ*^, then the power, *θ*, and proportionality constant, *γ*, can be determined for each network by linear regression on data with product degrees less than the cutoff (Table 1). We find that all powers, *θ*, are very close to one, which is consistent with a probability function that is linear in each degree [23,24]. The proportionality constants *γ *determined from the regressions can also be calculated from normalizations via *γ*(cal) = *E*/Σ_*i*<*j*_(*k*_*i*_*k*_*j*_), where *E *is the total number of interactions in the network and the summation is over all pairs of proteins. We find that the fitted and calculated proportionality constants are in good agreement (Table 1). Therefore, not only is degree-weighted behaviour evident in the networks but this property can straightforwardly be extracted, and modelled by *P*_*ij *_= *γk*_*i*_*k*_*j*_, where the proportionality constant *γ *is determined from the degrees of the proteins.

Having demonstrated that PPI networks exhibit degree-weighted behaviour up to a certain value of the degree product *k*_1_*k*_2_, we turn our attention to these nonconforming regions of the networks. Of the networks analyzed here, only that of *P. falciparum *(Figure 1i) shows a degree-weighted tendency throughout. In terms of the number of interactions, this network is the second smallest only to that of the high-confidence network of *Caenorhabditis elegans *(Figure 1h), which shows more consistent behaviour in the high-degree product range than the other networks. However, there does not seem to be any association between levels of consistency and the sizes of the PPI networks. The nature of the deviations from degree-weighted behaviour is similar in all networks (Figure 1) and consists of a levelling off in values of *P*(*k*_1_, *k*_2_) together with increased variability. An important observation is that the probabilities of interaction in these high-degree areas are still quite high when compared to the well-behaved, lower-degree interaction regions. Thus, even though the high-degree nodes (or hub proteins) do not seem to obey degree-weighted behaviour, they still prefer to interact with each other rather than with lower-degree proteins. These findings are similar to that reported previously, in that the hub proteins act somewhat differently to the remainder of the proteins [25]. However, in contrast, we find that interactions between hub proteins have high probability compared to an interaction between low-degree nodes. It has been commonly accepted that hubs in a network avoid each other [25], however, we do not find this to be so.

### Impact of degree-weighted behaviour upon network topology

**Figure 2:**
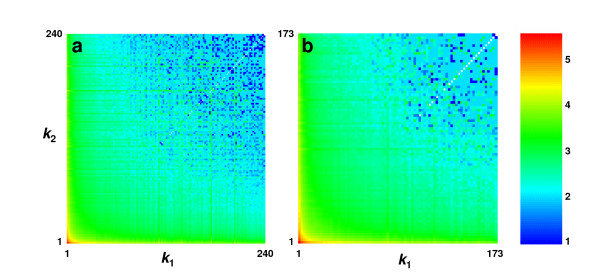
**Distance profiles in two protein-protein interaction networks**. **a**, *Homo sapiens*; **b**, *Drosophila melanogaster*. Distances shown as average shortest path lengths *L*(*k*_1_, *k*_2_) between proteins of degrees *k*_1 _and *k*_2_.

Further insight regarding the hub-hub connectivity, as well as the overall topology of the PPI networks, can be gained by determining the average path lengths *L*(*k*_1_, *k*_2_) between proteins of degrees *k*_1 _and *k*_2_. We investigate such maps for each of the PPI networks studied here. Figure 2 illustrates maps for the networks of *Homo sapiens *and *Drosophila melanogaster*, while maps for the remaining networks are provided in Additional file 2. As expected, the lowest-degree proteins are typically separated by the largest number of links, and the distance between proteins is decreased as either, or both, of their degrees are increased. For *H. sapiens*, this trend extends through to the high-degree interacting proteins, as most of the hubs are separated by only one or two links, indicating that they do not avoid each other. Therefore, this network incorporates a scale-rich element [26] as well as a hierarchical nature in that the hub proteins are somewhat interconnected and generally closer to higher-degree proteins. For *D. melanogaster*, the hubs appear slightly less clustered than in *H. sapiens*, with separations of mostly one, two, and three links. If most of the shortest paths between proteins traverse the interconnected hub areas, then this explains why the overall average path length for the *H. sapiens *network (4.28) is smaller than for the *D. melanogaster *network (4.41) even though the former is much larger. Maps for all PPI networks studied here show similar features. The only real differences are in the connectivities of the high-degree proteins, which can be enhanced (*H. sapiens*, *E. coli*, *C. elegans*, *P. falciparum*) or slightly diminished (*D. melanogaster*, *Saccharomyces cerevisiae*).

The path length maps clearly demonstrate that almost every high-degree protein has another high-degree protein located within one or two steps. This implies that any existing modules that incorporate one or more high-degree proteins are very likely to overlap or neighbour each other. As such, it is doubtful that *isolated *modules, or dense clusters, will contain high-degree proteins. Rather, they might contain proteins of more modest degree. However, the maps also indicate that any protein is, on the average, within three steps of a hub. Therefore, isolated complexes, if they exist, are likely to be few steps away from a high-degree protein. The observed trends in degree-weighted behaviour and shortest path lengths suggests that the PPI networks are extremely dense in their core, or interconnected hub region, and become somewhat sparser as the number of steps from the core is increased. Therefore, if any concentrated clusters are identified by some graph theoretical criterion, then there are probably many other complexes satisfying, or very nearly satisfying, this criterion. Thus, the concept of an isolated module becomes indistinct.

### Analogy between degree-weighted connectivity and randomness

**Figure 3:**
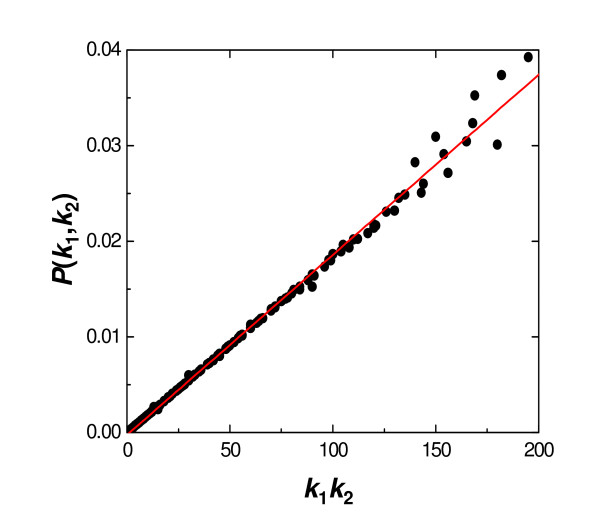
**Degree weighted connectivity in the Erdös-Rényi random graph model equivalent to the PPI network of *P. falciparum *(1304 nodes, 2745 edges)**. Probabilities of interaction *P*(*k*_1_, *k*_2_) are calculated for 10^4 ^realizations, which are then averaged over the number of simulated networks that contain nodes of degree *k*_1 _*and k*_2_.

To illustrate the concept of inherent randomness in networks displaying degree-weighted behaviour, we show how Erdös-Rényi (ER) random graphs [10,11] also exhibit a degree-weighted characteristic. ER random networks have degree distributions that are Poissonian about the average degree and are, therefore, different from those of PPI networks, which show power-law scaling. Nonetheless, examination of the connectivity profile in ER networks will shed light on the interpretation of degree-weighted behaviour. The model we studied here is an ER random graph equivalent to the PPI network of *P. falciparum*, i.e., the probability of any edge is determined from the number of nodes and edges in the network of *P. falciparum*. We analyzed the extent of degree-weighted behaviour in this ER model by computing the probabilities *P*(*k*_1_, *k*_2_) of interaction between two proteins of degrees *k*_1 _and *k*_2 _over 10^4 ^realizations of the network. However, each probability *P*(*k*_1_, *k*_2_) was only averaged over the number of generated networks that contain nodes of degree *k*_1 _*and k*_2_. The reason for this is that nodes of higher degree may not occur in every realization. The resulting relationship between the probability of interaction *P*(*k*_1_, *k*_2_) and the degree product *k*_1_*k*_2 _is shown in Figure 3. As expected, this plot clearly suggests that ER random networks are inherently degree weighted, i.e., *P*(*k*_1_, *k*_2_) ∝ *k*_1_*k*_2_. The results further suggest that degree-weighted behaviour may be an indication, or property, of randomness. The ramifications of these findings are not immediately obvious and further analysis is required to comprehensively assess whether PPI networks incorporate random elements. However, our preliminary analysis indicates that randomness may play a significant role in these biological networks.

## Discussion and Conclusion

The degree-weighted nature of PPI networks as well as the hub grouping present a quandary in that it implies that the assembly of these networks may be less biologically guided and more probabilistic in nature. One reason for this may be that high-throughput methods make little assumption about a protein's locality in the cell and therefore allow for more interactions than might be observed *in vivo*. In fact, only 40–50% of the identified interactions from high-throughput yeast two-hybrid (Y2H) analyses of *S. cerevisiae *were between proteins occurring in the same cellular compartment [27,28]. However, the Yeast-CORE PPI network, which is considered to be high confidence and has a high conservation of interactions between proteins of the same compartment [29], exhibits a high level of degree-weighted behaviour (Figure 1d). Another consideration is that the various approaches to identify protein-protein interactions unintentionally bias their collation from the different functional and cell component categories [28]. However, all the PPI networks studied here show similar degree-weighted connectivity even though five of them (Figures 1b (*D. melanogaster*), 1e (*S. cerevisiae*), 1g–h (*C. elegans*), and 1i (*P. falciparum*)) are almost completely determined from Y2H screens, while the remaining four are compiled from a variety of experimental sources (see Additional file 1).

It could also be that PPI networks determined from high-throughput methods contain non-specific interactions. Such variability is not unexpected considering the large amount of irreproducibility of once-identified interactions [30]. In such a case, we might expect to see similar probabilistic behaviour as that observed here. Contrary to this, though, the high-confidence network of *C. elegans *[30], which contains interactions found in three independent repeated experiments, exhibits clear degree-weighted characteristics (Figure 1h).

Obviously, protein-protein interactions are necessary for a myriad of biological processes, however, if the event is "controlled" by other time- and location-dependent processes, the actual binding or interaction could be of secondary importance. If degree-weighted behaviour is observed in a network, i.e., if protein interactions appear probabilistic, an analysis of expected binding events will determine whether the observed binding events are guided by their interactions or just by their ability to bind. This will greatly enhance the capability of interpreting and extracting biological information from protein-protein interaction networks. The findings presented here provide a cautionary note on the biological interpretation of large PPI networks. One interpretation of the observed degree-weighted networks is that we are witnessing the ability to bind, and not necessarily what connections/interactions are actually present in the cell. The true biological connections that are used in a pathway or biological process cannot be back-engineered from this type of data without taking into account a degree-weighted model, and hence the topological study of a degree-weighted network requires a more refined methodology to extract biological information about pathways, modules, or other inferred relationships among proteins. *A priori *knowledge of a protein's degree or connectivity is not available, however, algorithms to predict this [31,32], as well as their interactions [32-34], are being developed. Whether application of these predictive algorithms on genomic scales yield degree-weighted networks remains to be seen, and may even serve as a test for the verity of the resultant network topologies.

Further insight into the degree-weighted nature of PPI networks may be obtained from analyses of the interacting protein pairs at more elementary levels. An avenue for this dissection has been to characterize the structural and functional domains present in each protein [35,36] and subsequently identify consistent signatures, i.e., pairs of domains that are more likely to be involved in binding [37,38]. In this way, domain-domain interaction (DDI) networks can be derived and then compared against PPI networks to see if they have similar topological properties such as degree-weighted behaviour. If, for example, degree-weighted behaviour is not observed in DDI networks, then one would anticipate consistent precepts for the allowed interactions, thereby allowing for alternative, and more insightful, analyses of PPI networks.

One utility of knowing that a network is degree-weighted is to use the probabilistic interpretation to find nodes that deviate from degree-weighted probability. Such nodes would represent a potential network that is biologically deterministic by its protein-protein interactions alone. For example, clusters of low-degree proteins might imply selective complex formation, and hubs found to be isolated from other high-degree proteins may represent important bottlenecks.

## Authors' contributions

All authors contributed to the design and coordination of the study. JI performed the computational implementations and prepared the original draft, which was revised by AW and JR. All authors read and approved the final manuscript.

## Supplementary Material

Additional file 1 Descriptions and sources of the protein-protein interaction networks used in this work. Click here for file

Additional file 2 **Supplementary Figure – Distance profiles in protein-protein interaction networks**. **a-c**, *Saccharomyces cerevisiae *(yeast): Yeast-DIP, Yeast-CORE, Yeast-Y2H; **d**, *Escherichia coli *(bacterium); **e**-**f**, *Caenorhabditis elegans *(nematode): Worm-Y2H, Worm-CORE; **g**, *Plasmodium falciparum *(malaria-causing parasite). Distances shown as average shortest path lengths *L*(*k*_1_, *k*_2_) between proteins of degrees *k*_1 _and *k*_2_. Click here for file
