# Supplementary material for: Evidence of probabilistic behaviour in protein interaction networks
Source: BMC Syst Biol. 2008 Jan 31;2:11. doi: 10.1186/1752-0509-2-11 (PMC2267158; doi:10.1186/1752-0509-2-11)
Supplement: Additional file 1 — Descriptions and sources of the protein-protein interaction networks used in this work. [file 1752-0509-2-11-S1.pdf]

Evidence of probabilistic behaviour in protein interaction networks

Supplementary Information

Protein-protein interaction networks

We studied a total of nine PPI networks comprising six unique organisms: *Homo sapiens* (human), *Drosophila melanogaster* (fruit fly), *Saccharomyces cerevisiae* (yeast), *Escherichia coli* (bacterium), *Caenorhabditis elegans* (nematode), and *Plasmodium falciparum* (malaria-causing parasite). In all cases, protein self-interactions were not considered and an undirected nature for the networks was assumed, i.e., an interaction A−B is equivalent to the interaction B−A.

One PPI network of *H. sapiens* was analyzed and this was downloaded from the Human Protein Reference Database (HPRD, http://www.hprd.org) [1, 2]. This collection of interactions is manually extracted from the literature.

For *E. coli* and *D. melanogaster*, one PPI network of each was studied and both were derived from the Database of Interacting Proteins (DIP, http://dip.doe-mbi.ucla.edu) [3], which curates a diverse body of experimentally-determined interactions for a range of organisms. The list of sources is too vast to describe here but includes data from high-throughput methods, including Y2H [4, 5], protein microarrays [6] and mass spectrometric analysis of highly purified multi-protein complexes [7, 8] as well as from

analysis of protein complexes stored in the Protein Data Bank [9]. For *E. coli*, only interactions between proteins belonging to the K12 strain were considered and these were labelled (or relabelled) by their most recent Swiss-Prot Accession Numbers [10]. *D. melanogaster* proteins were labelled (or relabelled) by their most up-to-date GenInfo identifiers using the National Center for Biotechnology Information (NCBI) Sequence Revision History tool (http://www.ncbi.nlm.nih.gov/entrez/sutils/girevhist.cgi). The use of a particular strain and updating of protein labels to their more recent forms ensures that the PPI networks are as biologically consistent as possible.

Three PPI networks of *S. cerevisiae* were analyzed. Two of these, hereafter referred to as *Yeast-DIP* and *Yeast-CORE*, were downloaded from DIP. The *Yeast-CORE* network is a subset of the *Yeast-DIP* network in that the *Yeast-CORE* set is said to include a more reliable set of interactions that have been verified using the Paralogous Verification Method and the Expression Profile Reliability Index [11]. In both cases, proteins were labelled (or relabelled) using up-to-date Swiss-Prot accession numbers and only interactions between proteins of the strain S288C were considered. The third *S. cerevisiae* network used in this work includes only interactions determined by Y2H screens [5, 12] for a variant of the S288C strain [13]. This network is designated as *Yeast-Y2H*.

Two PPI networks of *C. elegans* were used in this study and both were determined from equivalent Y2H screens [14]. The first, labelled *Worm-Y2H*, is a compilation of all identified interactions and the second, labelled *Worm-CORE*, contains a high-confidence subset of interactions that were consistently identified in three repeated experiments.

The final PPI network analyzed here is that of the malaria-causing parasite *P. falciparum*, which was taken from Y2H experimental data [15].

Supplementary References

1. Mishra GR, Suresh M, Kumaran K, Kannabiran N, Suresh S, Bala P, Shivakumar K, Anuradha N, Reddy R, Raghavan TM, Menon S, Hanumanthu G, Gupta M, Upendran S, Gupta S, Mahesh M, Jacob B, Mathew P, Chatterjee P, Arun KS, Sharma S, Chandrika KN, Deshpande N, Palvankar K, Raghavnath R, Krishnakanth R, Karathia H, Rekha B, Nayak R, Vishnupriya G, Kuma HG, Nagini M, Kumar GS, Jose R, Deepthi P, Mohan SS, Gandhi TK, Harsha HC, Deshpande KS, Sarker M, Prasad TS, Pandey A: **Human protein reference database--2006 update**. *Nucleic acids research* 2006, **34**(Database issue):D411-414.
2. Peri S, Navarro JD, Amanchy R, Kristiansen TZ, Jonnalagadda CK, Surendranath V, Niranjan V, Muthusamy B, Gandhi TK, Gronborg M, Ibarrola N, Deshpande N, Shanker K, Shivashankar HN, Rashmi BP, Ramya MA, Zhao Z, Chandrika KN, Padma N, Harsha HC, Yatish AJ, Kavitha MP, Menezes M, Choudhury DR, Suresh S, Ghosh N, Saravana R, Chandran S, Krishna S, Joy M, Anand SK, Madavan V, Joseph A, Wong GW, Schiemann WP, Constantinescu SN, Huang L, Khosravi-Far R, Steen H, Tewari M, Ghaffari S, Blobe GC, Dang CV, Garcia JG, Pevsner J, Jensen ON, Roepstorff P, Deshpande KS, Chinnaiyan AM, Hamosh A, Chakravarti A, Pandey A: **Development of human protein reference database as an initial platform for approaching systems biology in humans**. *Genome research* 2003, **13**(10):2363-2371.
3. Salwinski L, Miller CS, Smith AJ, Pettit FK, Bowie JU, Eisenberg D: **The Database of Interacting Proteins: 2004 update**. *Nucleic acids research* 2004, **32**(Database issue):D449-451.
4. Uetz P, Giot L, Cagney G, Mansfield TA, Judson RS, Knight JR, Lockshon D, Narayan V, Srinivasan M, Pochart P, Qureshi-Emili A, Li Y, Godwin B, Conover D, Kalbfleisch T, Vijayadamodar G, Yang M, Johnston M, Fields S, Rothberg JM: **A comprehensive analysis of protein-protein interactions in Saccharomyces cerevisiae**. *Nature* 2000, **403**(6770):623-627.
5. Ito T, Chiba T, Ozawa R, Yoshida M, Hattori M, Sakaki Y: **A comprehensive two-hybrid analysis to explore the yeast protein interactome**. *Proceedings of the National Academy of Sciences of the United States of America* 2001, **98**(8):4569-4574.
6. Zhu H, Bilgin M, Bangham R, Hall D, Casamayor A, Bertone P, Lan N, Jansen R, Bidlingmaier S, Houfek T, Mitchell T, Miller P, Dean RA, Gerstein M, Snyder M: **Global analysis of protein activities using proteome chips**. *Science (New York, NY* 2001, **293**(5537):2101-2105.
7. Gavin A-C, Bosche M, Krause R, Grandi P, Marzioch M, Bauer A, Schultz J, Rick JM, Michon AM, Cruciat CM, Remor M, Höfert C, Schelder M, Brajenovic M, Ruffner H, Merino A, Klein K, Hudak M, Dickson D, Rudi T, Gnau V, Bauch A, Bastuck S, Huhse B, Leutwein C, Heurtier MA, Copley RR, Edelmann A, Querfurth E, Rybin V, Drewes G, Raida M, Bouwmeester T, Bork P, Seraphin B, Kuster B, Neubauer B, Superti-Furga G: **Functional organization of the yeast proteome by systematic analysis of protein complexes**. *Nature* 2002, **415**(6868):141-147.

8. Ho Y, Gruhler A, Heilbut A, Bader GD, Moore L, Adams SL, Millar A, Taylor P, Bennett K, Boutilier K, Yang L, Wolting C, Donaldson I, Schandorff S, Shewnarane J, Vo M, Taggart J, Goudreault M, Muskat B, Alfarano C, Dewar D, Lin Z, Michalickova K, Willems AR, Sassi H, Nielsen PA, Rasmussen KJ, Andersen JR, Johansen LE, Hansen LH, Jespersen H, Podtelejnikov A, Nielsen E, Crawford J, Poulsen V, Sørensen BD, Matthiesen J, Hendrickson RC, Gleeson F, Pawson T, Moran MF, Durocher D, Mann M, Hogue CW, Figeys D, Tyers M: **Systematic identification of protein complexes in Saccharomyces cerevisiae by mass spectrometry**. *Nature* 2002, **415**(6868):180-183.
9. Westbrook J, Feng Z, Jain S, Bhat TN, Thanki N, Ravichandran V, Gilliland GL, Bluhm W, Weissig H, Greer DS, Bourne PE, Berman HM: **The Protein Data Bank: unifying the archive**. *Nucleic acids research* 2002, **30**(1):245-248.
10. Boeckmann B, Bairoch A, Apweiler R, Blatter MC, Estreicher A, Gasteiger E, Martin MJ, Michoud K, O'Donovan C, Phan I, Pilbout S, Schneider M: **The SWISS-PROT protein knowledgebase and its supplement TrEMBL in 2003**. *Nucleic acids research* 2003, **31**(1):365-370.
11. Deane CM, Salwinski L, Xenarios I, Eisenberg D: **Protein interactions: two methods for assessment of the reliability of high throughput observations**. *Mol Cell Proteomics* 2002, **1**(5):349-356.
12. Ito T, Tashiro K, Muta S, Ozawa R, Chiba T, Nishizawa M, Yamamoto K, Kuhara S, Sakaki Y: **Toward a protein-protein interaction map of the budding yeast: A comprehensive system to examine two-hybrid interactions in all possible combinations between the yeast proteins**. *Proceedings of the National Academy of Sciences of the United States of America* 2000, **97**(3):1143-1147.
13. Hudson JR, Jr., Dawson EP, Rushing KL, Jackson CH, Lockshon D, Conover D, Lanciault C, Harris JR, Simmons SJ, Rothstein R, Fields S: **The complete set of predicted genes from Saccharomyces cerevisiae in a readily usable form**. *Genome research* 1997, **7**(12):1169-1173.
14. Li S, Armstrong CM, Bertin N, Ge H, Milstein S, Boxem M, Vidalain PO, Han JD, Chesneau A, Hao T, Goldberg DS, Li N, Martinez M, Rual JF, Lamesch P, Xu L, Tewari M, Wong SL, Zhang LV, Berriz GF, Jacotot L, Vaglio P, Reboul J, Hirozane-Kishikawa T, Li Q, Gabel HW, Elewa A, Baumgartner B, Rose DJ, Yu H, Bosak S, Seguerra R, Fraser A, Mango SE, Saxton WM, Strome S, Van Den Heuvel S, Piano F, Vandenhaute J, Sardet C, Gerstein M, Doucette-Stamm L, Gunsalus KC, Harper JW, Cusick ME, Roth FP, Hill DE, Vidal M: **A map of the interactome network of the metazoan C. elegans**. *Science (New York, NY* 2004, **303**(5657):540-543.
15. LaCount DJ, Vignali M, Chettier R, Phansalkar A, Bell R, Hesselberth JR, Schoenfeld LW, Ota I, Sahasrabudhe S, Kurschner C, Fields S, Hughes RE: **A protein interaction network of the malaria parasite Plasmodium falciparum**. *Nature* 2005, **438**(7064):103-107.
