# Supplementary material for: Evidence of probabilistic behaviour in protein interaction networks
Source: BMC Syst Biol. 2008 Jan 31;2:11. doi: 10.1186/1752-0509-2-11 (PMC2267158; doi:10.1186/1752-0509-2-11)
Supplement: Additional file 2 — Supplementary Figure – Distance profiles in protein-protein interaction networks. a-c, Saccharomyces cerevisiae (yeast): Yeast-DIP, Yeast-CORE, Yeast-Y2H; d, Escherichia coli (bacterium); e-f, Caenorhabditis elegans (nematode): Worm-Y2H, Worm-CORE; g, Plasmodium falciparum (malaria-causing parasite). Distances shown as average shortest path lengths L(k1, k2) between proteins of degrees k1 and k2. [file 1752-0509-2-11-S2.pdf]

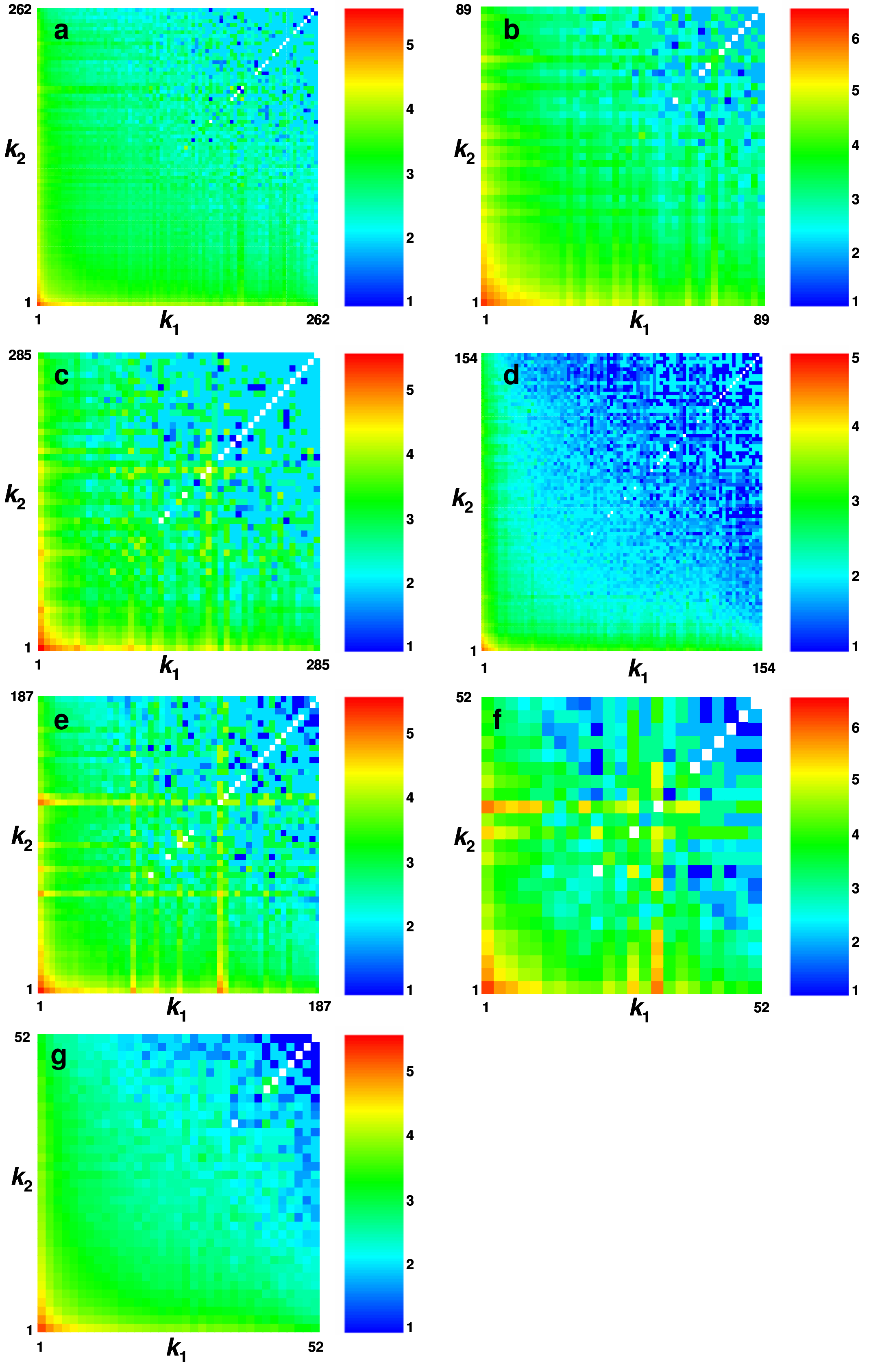

Supplementary Figure – Distance profiles in protein-protein interaction networks
a-c, *Saccharomyces cerevisiae* (yeast): Yeast-DIP, Yeast-CORE, Yeast-Y2H; **d**, *Escherichia coli* (bacterium); **e-f**, *Caenorhabditis elegans* (nematode): Worm-Y2H, Worm-CORE; **g**, *Plasmodium falciparum* (malaria-causing parasite). Distances shown as average shortest path lengths $L(k_1,k_2)$ between proteins of degrees k_1 and k_2.
